# Regulation of Inflammatory Response by Transmembrane Adaptor Protein LST1

**DOI:** 10.3389/fimmu.2021.618332

**Published:** 2021-04-27

**Authors:** Matej Fabisik, Jolana Tureckova, Nataliia Pavliuchenko, Jarmila Kralova, Jana Balounova, Kristina Vicikova, Tereza Skopcova, Frantisek Spoutil, Jana Pokorna, Pavla Angelisova, Bernard Malissen, Jan Prochazka, Radislav Sedlacek, Tomas Brdicka

**Affiliations:** ^1^ Laboratory of Leukocyte Signalling, Institute of Molecular Genetics of the Czech Academy of Sciences, Prague, Czechia; ^2^ Faculty of Science, Charles University, Prague, Czechia; ^3^ Laboratory of Transgenic Models of Diseases, Institute of Molecular Genetics of the Czech Academy of Sciences, Vestec, Czechia; ^4^ Czech Centre for Phenogenomics, Institute of Molecular Genetics of the Czech Academy of Sciences, Vestec, Czechia; ^5^ Centre d’Immunophénomique, Aix Marseille Université, INSERM, CNRS, Marseille, France

**Keywords:** LST1, inflammation, colitis, inflammatory bowel disease, myeloid cells

## Abstract

LST1 is a small adaptor protein expressed in leukocytes of myeloid lineage. Due to the binding to protein tyrosine phosphatases SHP1 and SHP2 it was thought to have negative regulatory function in leukocyte signaling. It was also shown to be involved in cytoskeleton regulation and generation of tunneling nanotubes. *LST1* gene is located in MHCIII locus close to many immunologically relevant genes. In addition, its expression increases under inflammatory conditions such as viral infection, rheumatoid arthritis and inflammatory bowel disease and its deficiency was shown to result in slightly increased sensitivity to influenza infection in mice. However, little else is known about its role in the immune system homeostasis and immune response. Here we show that similar to humans, LST1 is expressed in mice in the cells of the myeloid lineage. *In vivo*, its deficiency results in alterations in multiple leukocyte subset abundance in steady state and under inflammatory conditions. Moreover, LST1-deficient mice show significant level of resistance to dextran sodium sulphate (DSS) induced acute colitis, a model of inflammatory bowel disease. These data demonstrate that LST1 regulates leukocyte abundance in lymphoid organs and inflammatory response in the gut.

## Introduction

Leukocyte-specific transcript 1 protein (LST1) is a small, 97 amino acid long, transmembrane adaptor protein. It is composed of a very short extracellular segment with a dimerization cysteine, a single transmembrane domain, immediately followed by a palmitoylation site, and a larger cytoplasmic tail with two immunoreceptor tyrosine-based inhibitory motifs (ITIM). Despite of its small size, at least 16 *LST1* splice variants of various length (transmembrane and soluble isoforms) were described in human mRNA. However, only one of these variants LST1/A has been detected at the protein level (1–4). The role of this extensive splicing is not known. It has been speculated that it might function as a transcription and translation regulation tool (5). Interestingly, only two RNA splice forms have been detected in mouse (2). In this report, we will refer to the protein expressed from human and murine *LST1* genes only as LST1.

Our previous analysis of LST1 expression pattern by in-house generated monoclonal antibody LST1/02 recognizing human but not murine LST1 revealed its expression exclusively in leukocytes of the myeloid lineage (macrophages, dendritic cells, monocytes, granulocytes) and in related cell lines (U-937, THP-1) (1). However, there is a discrepancy between these results and results obtained with another monoclonal antibody 7E2, which showed expression of LST1 also in lymphoid (Jurkat, B cells) and non-hematopoietic cells (HeLa, Capan-1, HepG2) (3). LST1 expression appears to be regulated during inflammation. Increased expression of LST1 mRNA isoforms was detected in cell lines after treatment with pro-inflammatory compounds (LPS, TNFα). Its expression was also elevated in histological colon samples from patients with inflammatory bowel disease (IBD) (6) and in the synovial fluid of patients with rheumatoid arthritis (7).

LST1 is coded by *LST1* gene (also known as *B144*) localized in *MHCIII* locus. This genomic site harbors many immunologically important genes, such as genes coding for Lymphotoxin-β, Tumor Necrosis Factor α, several complement proteins and others. High LST1 expression in leukocytes together with localization of its gene in one of the immunologically most important loci, raises a question about the function of LST1 in the immune system (8, 9). Previous work from our laboratory demonstrated that ITIM motifs in LST1 bind phosphatases SHP1 and SHP2 and suggested that it is a negative regulator of signaling in myeloid cells, although the processes that LST1 regulates *in vivo* were not defined (1).

In HeLa cells, overexpression of LST1 induced formation of tunneling nanotubes *via* interaction with RalA–M-Sec–exocyst complex, and transfer of MHC class I molecules through these nanotubes between the cells (10–12). In a genomic study, *Lst1* was identified as a gene connected to host response to influenza virus (13). This was further corroborated by a subsequent study showing that LST1-deficient mice display higher susceptibility to influenza infection when compared to the wild type mice (14). Increased expression of *LST1* in tissues affected by IBD or rheumatoid arthritis suggests that it may also be involved in other inflammatory conditions. In this work we describe basic features of LST1 deficient mice and analyze the role of LST1 in the dextran sodium sulphate (DSS)-induced colitis, a mouse model of IBD. We show that the LST1 deficiency results in alterations in innate leukocyte subset composition and in milder progress of DSS-induced colitis, demonstrating LST1 involvement in the regulation of leukocyte homeostasis and inflammation.

## Materials and Methods

### Mice

LST1-deficient mouse strain LST1^tm1(KOMP)Vlcg^ on C57Bl/6J genetic background (abbreviated as *Lst1^-/-^*) was obtained from International Knockout Mouse Consortium. These animals were crossed to C57Bl/6J mice from Animal facility of the Institute of Molecular Genetics to obtain heterozygotes *Lst1^-/-^* x C57Bl/6J. Their homozygote offspring were used as littermates for comparative experiments at the age of 6 - 10 weeks. Animal experiments were approved by the Animal Care and Use Committee of the Institute of Molecular Genetics and were in agreement with local legal requirements and ethical guidelines.

### Primary Cell Isolation and Activation

Animals were sacrificed by cervical dislocation and single cell suspensions were prepared. Lymph node and splenic cell suspensions were prepared by pressing the lymph node or spleen tissue through 42 μm cell strainer. Bone marrow cells were isolated by flushing femurs cut at the extremities with cold PBS/2% FCS using syringe and 30 Gauge needle. Erythrocytes were removed by lysis in ACK buffer (150 mM NH_4_Cl, 0.1 mM EDTA (disodium salt), 1 mM KHCO_3_). Colon lamina propria cells were isolated from entire colon. The colon was opened longitudinally, washed with cold PBS and then rocked for 1 hour in 10 ml solution of 5mM EDTA/2% FCS/HBSS without Ca^2+^ and Mg^2+^ at 4°C followed by washing with 10 ml HBSS/2% FCS. Colon was then cut to 3 mm pieces and digested with the solution containing collagenase II (2 mg/ml, Gibco #17101-015, CAS No. 9001-12-1) and DNase I (0.5mg/ml, Sigma powder DN25-100MG; CAS No. 9003-98-9) in DMEM/2% FCS with shaking (37°C, 2 × 30 min). After each digestion round, tissues were poured onto petri dish and triturated gently with plastic Pasteur pipette in order to obtain the lamina propria cells. Cell suspension was filtered through 100 µm Sysmex filters, centrifuged, resuspended in 5 ml of PBS/2% FCS and kept on ice. After the second round of digestion, cells were pooled together, centrifuged, resuspended in PBS/2% FCS and filtered again through 50 µm Sysmex filters to obtain single cell suspension of colon lamina propria cells. For murine colonic epithelial cell isolation, colon was opened longitudinally and washed vigorously in PBS on ice. Then it was cut to shorter fragments and incubated in 20 ml pre-heated HBSS, 3% FBS, 2 mM EDTA twice at 37°C. The suspension with released cells was then collected and filtered through 100 µm filter. Filtered cells were resuspended in 1 ml (1 min, 37°C) TrypLE™ Express Enzyme (Thermo Fischer Scientific, 12605010), resuspended by pipetting and filtered again through 40 µm filter into 15 ml tubes. TrypLE™ enzymes were neutralized by washing filter with 3 ml of HBSS (without Ca^2+^ and Mg^2+^), 3% FBS, 2 mM EDTA (pH 8.0, ice cold). Cells were centrifuged for 10 min (4°C, 300 × g), stained with CD45 and EpCAM fluorescent antibodies and sorted on BD FACSAria™ cell sorter as CD45^-^ EpCAM^++^. Bone marrow derived dendritic cells (BMDC) and bone marrow derived macrophages (BMDM) were generated from isolated mouse bone marrow cells by culturing in IMDM culture media supplemented with 10% FCS and 3% supernatant from J558 cells containing GM-CSF for 10 days (BMDC) or 5% supernatant from CMG 14-12 cells containing M-CSF for 7 days (BMDM) as described in detail here (15).

### Glycosylation Assay

BMDM were plated in a 6-well plate at 2 × 10^6^ cells per well and treated with Tunicamycin (Sigma) - 0.5 µg/ml and 1 µg/ml for 24 hours followed by lysis (300 µl 2× concentrated SDS-PAGE sample buffer). Lysed samples were sonicated, heated to 95°C for 10 minutes and analyzed by immunoblotting under non-reducing conditions.

### Cell Activation

BMDM and BMDC were cultured overnight in DMEM without M-CSF/GM-CSF in a 12-well tissue culture plate (10^6^ cells per well) and then incubated for indicated time-points with IFNγ (Peprotech) – 50 ng/ml, TNFα (Peprotech) – 20 ng/ml, LPS (Sigma) – 100 ng/ml and PolyI:C (Invivogen, low molecular weight) – 20 µg/ml, GM-CSF – 3% supernatant from J558 cells. Subsequently, cells were lysed in 2 × concentrated SDS-PAGE sample buffer (128 mM Tris pH 6.8, 10% glycerol, 4% SDS, 1% DTT) and subjected to immunoblotting with indicated antibodies.

### Antibodies

Flow cytometry antibodies are listed in **Supplementary Table S1**. LST1/06 mouse mAb recognizing murine LST1 on Western blots and in immunoprecipitation was generated by immunization of *Lst1^-/-^* mice with recombinant intracellular domain of murine LST1 (starting at Cys40) produced in *Escherichia coli*. After immunization, splenocytes were fused with Sp2/0 myeloma cells and antibody producing hybridomas were cloned by limiting dilution. Antibodies used for Western blot detection: SHP-1 (SH-PTP1 Antibody, C-19, Santa Cruz Biotechnology), SHP-2 (SH-PTP2 N-16, Santa Cruz Biotechnology), GAPDH (Anti-GAPDH antibody produced in rabbit, Sigma-Aldrich), IκBα (Cell Signaling Technology, #9242). Western blot ECL signals were detected with Azure c300 imaging system (Azure Biosystems) and quantified using AIDA image analyzer software (Elysia-raytest, Straubenhardt, Germany).

### Flow Cytometry

Cells of bone marrow, spleen and lymph nodes were incubated with fluorescently labeled antibodies and Fc-receptor blocking antibody (2.4G2) and analyzed on BD™ LSRII flow cytometer (Becton Dickinson). Staining was divided into two sets – A (CD3, CD4, CD8, CD19, NK1.1), B (CD11b, CD11c, F4/80, Ly6C, Ly6G). Cell populations were gated as: CD4 T cells – CD3^+^ CD4^+^; CD8 T cells – CD3^+^ CD8^+^; B cells – CD19^+^ CD3^-^; NK cells – NK1.1^+^ CD3^-^; NK1.1^+^CD3^+^; Macrophages – CD11b^low^ F4/80^+^; Monocytes – CD11b^+^ Ly6C^hi^ Ly6G^-^; Neutrophils – CD11b^+^ Ly6C^med^ Ly6G^+^; Dendritic cells – CD11b^+^ CD11c^+^. Colon leukocytes were measured on BD™ FACSymphony flow cytometer (Becton Dickinson) according to International Mouse Phenotyping Consortium standards^1^ and gated as CD45+ cells. Staining was divided into two sets – A (CD49d, CD4, Klrg1, CD44, CD8a, γδTCR, NK1.1, GITR, CD25, CD62L), B (Ly6G, CD19, Ly6C, CD11b, CD21/CD35, F4/80, Bst2, NK1.1, CD23, CD11c, MHCII). Populations were gated as: CD4 T cells – CD5^+^ CD4^+^; CD8 T cells – CD5^+^ CD8^+^; B cells – CD19^+^ MHCII^+^; NK cells – CD5^-^ CD161^+^; NKT cells – CD5^+^ CD161^+^; Monocytes – CD11b^+^ Ly6C^+^ Ly6G^-^; Neutrophils – CD11b^+^ Ly6C^med^ Ly6G^+^; Macrophages – CD11b^+^ F4/80^+^; Dendritic cells – CD11b^+/-^ MHCII^+^ CD11c^+^. Data were analyzed in FlowJo™ software.

### Cytokine Detection by ELISA

Blood from tail vein was collected into EDTA tubes (EDTA 1000A, ref. 078035, KABE LABORTECHNIK, GmbH) and centrifuged (16 000 × g, 10 min, 4°C). Supernatant (plasma) was then transfered to fresh tubes and frozen in -80°C. Colons were homogenized in 400 µl RIPA lysis buffer (20 mM TRIS pH 7.5, 150 mM NaCl, 1% Nonidet P-40, 1% sodium deoxycholate, 0.1% SDS) containing 5 mM iodoacetamide (Sigma-Aldrich) and diluted Protease Inhibitor Cocktail Set III (Calbiochem, Merck) using AvansAHM1 Homogenizer on ice (10 s, maximum speed). The homogenates were cleared by centrifugation and frozen in -80°C. Concentrations of cytokines in blood plasma or colonic lysates were determined using IL-6 and TNF alpha Mouse Uncoated ELISA Kit with Plates (88-7324-22, 88-7064-22, Thermo Fischer Scientific) according to the manufacturer´s protocols.

### Bone MicroCT Analysis

Femurs of 28 mice were scanned *in-vivo* under 20% zoletile anesthesia in SkyScan 1176 (Bruker, Belgium) at the resolution of 8.67 µm with 0.5 mm aluminum filter (voltage = 50 kV, current = 250 µA, step rotation = 0.3°) with 180° rotation, and reconstructed in NRecon 1.6.10.4 (Bruker, Belgium) with parameters of smoothing = 4, ring artifact correction = 7, beam hardening correction = 23%, and spread of intensities from 0.007 to 0.11 AU. Only reconstructions without artifacts underwent analysis resulting in 42 femurs analyzed. The femurs were segmented in CT Analyzer 1.16.4.1 (Bruker, Belgium) and reoriented in DataViewer 1.5.2 (Bruker, Belgium). CT Analyzer was also used for semiautomatic selection of regions of interest (central diaphysis for cortical bone, and distal metaphysis for trabecular bone) and subsequent 2D and 3D analysis. Parameters describing bone volume, porosity, and structural complexity were recorded. Bone mineral density (BMD) and tissue mineral density (TMD) were recorded for trabeculae and corticals, respectively, based on the correlation with calibrated hydroxyapatite phantoms.

### Induction of Inflammatory Response *In Vivo*

For LPS challenge, mice were intraperitoneally injected with LPS (50 µg per animal). After 4 and 16 hours blood samples were collected and then mice were sacrificed by cervical dislocation and splenocyte subsets analyzed. To induce colitis with dextran sodium sulphate (DSS), littermates of *Lst1^-/-^* and WT mice (males, 8 weeks old) were fed with 2,5% DSS dissolved in drinking water for 6 days to induce acute colitis. Starting on day 7, DSS solution was changed for plain water. Parameters defining disease activity index, i.e. stool consistency, occult bleeding (Hemoccult Fecal Occult Blood Test, Beckman Coulter) and body weight on days 0, 2, 4, 5, 6, 8 were measured during the experiment. Mice in control group were administered only plain water. On day 8, mice were anesthetized with isoflurane (0.3L/min) for collection of blood sample from the eye vein and sacrificed. Colon length was recorded, organs were collected (spleen, mesenteric lymph nodes, blood sample and colon), processed into single cell suspensions and measured with BD™ FACSymphony flow cytometer. Disease Activity Index was evaluated as average of the three measured parameters (weight loss, stool consistency and occult bleeding index) on a particular day. Scoring values were normalized to day 0, which has an arbitrary value 0. Scoring system for body weight: 1, loss of 1% –5% weight; 2, loss of 5% –10% weight; 3, loss of 10% –20% weight; 4, 20% and higher body weight loss. Stool consistency: 0, well-formed stool pellets; 1, loose stool pellets; 2, pasty and semi-formed stools that did not adhere to the anus (mild diarrhea); 3, slimy stool (moderate diarrhea); 4, severe watery diarrhea that adhered to the anus and contained with blood. Occult bleeding: 0, no blood; 2, positive Hemoccult test, and 4, gross rectal bleeding.

### Histology

The entire colon was opened longitudinally, flushed with PBS and fixed in 4% neutral buffered formaldehyde for 48 hours as swiss roll. Samples were dehydrated, embedded in paraffin and sectioned at 5 μm and stained with hematoxylin-eosin. For histological evaluation, areas of inflammatory lesions were microscopically evaluated and quantified as described (16). Area of damaged colon epithelium was assessed in FIJI editor after recalculating pixel area to the real area in mm^2^ (4-5 animals per group).

### Migration Assay

Corning 6.5 mm diameter Transwell Permeable Supports with 8.0 µm pores were used. Inserts (upper well) with pores were coated with 100 µl fibronectin (50 µg/ml) for 2 hours at room temperature and washed two times with migration media (IMDM without antibiotics, 0.5% BSA). 10^5^ macrophages were added into the insert with migration media and 600 µl of migration media with CXCL12 (100 ng/ml) into the bottom well. After 2 hours at 37°C, 5% CO_2_ migrated cells were collected (attached cells were released with 0.2% EDTA in PBS). The collected cells were mixed with 10 µl of Flow Cytometry Absolute Count Standard™ (Bangs Laboratories) and counted in BD™ FACSymphony flow cytometer (Becton Dickinson).

### Quantitative Real-Time PCR

The total cellular RNA was isolated using Quick-RNA Miniprep Plus kit and transcribed into cDNA by RevertAid RT Reverse Transcription Kit, both used according to manufacturer’s instructions. The cDNA was then used as a template for real-time quantitative PCR performed on LightCycler 480 using SYBR Green I Master mix (Roche). Primers used in cytokine mRNA detection are listed in **Supplementary Table S2**.

### Statistics

Student´s t-test (two-tailed, unpaired) and One-way ANOVA with Tukey posttest for P-values and two-sided Grubb´s test for outlier recognition was performed in Graphpad Prism Software. Bars in figures represent mean ± SD, if not stated otherwise. P values lower than 0.05 are marked with asterisks as follows: * p ≤ 0.05, ** p ≤ 0.01, *** p ≤ 0.005, **** p ≤ 0.0001.

## Results

### Murine LST1 Is Glycosylated Transmembrane Adaptor Protein

**Figure 1:**
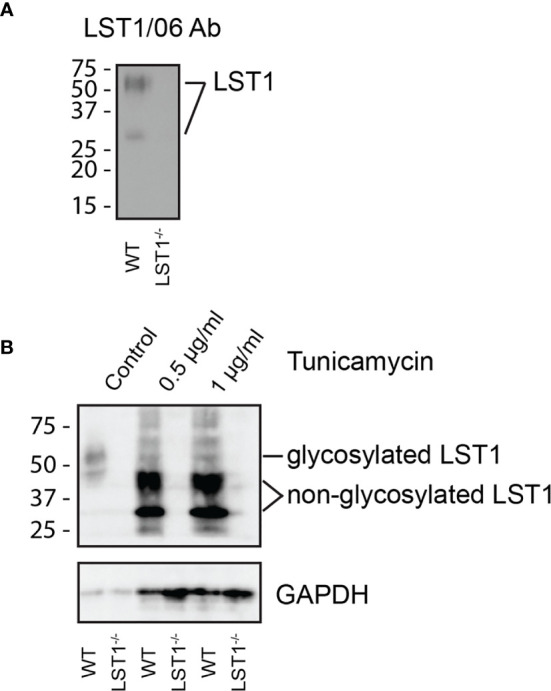
Murine LST1 is a glycoprotein recognized by a newly developed antibody LST1/06. **(A)** Detection of murine LST1 by LST1/06 antibody in the LST1/06 immunoprecipitates from WT and *Lst1^-/-^* BMDM in non-reduced samples (n=3). **(B)** Murine BMDM were cultured in the presence of tunicamycin (0.5 and 1 µg/ml). Cells were lysed and LST1 immunoprecipitated and analyzed by immunoblotting with LST1/06 antibody under non-reducing conditions (n=3). Note the electrophoretic mobility shift after tunicamycin treatment, which is indicative of LST1 glycosylation.

**Figure 2:**
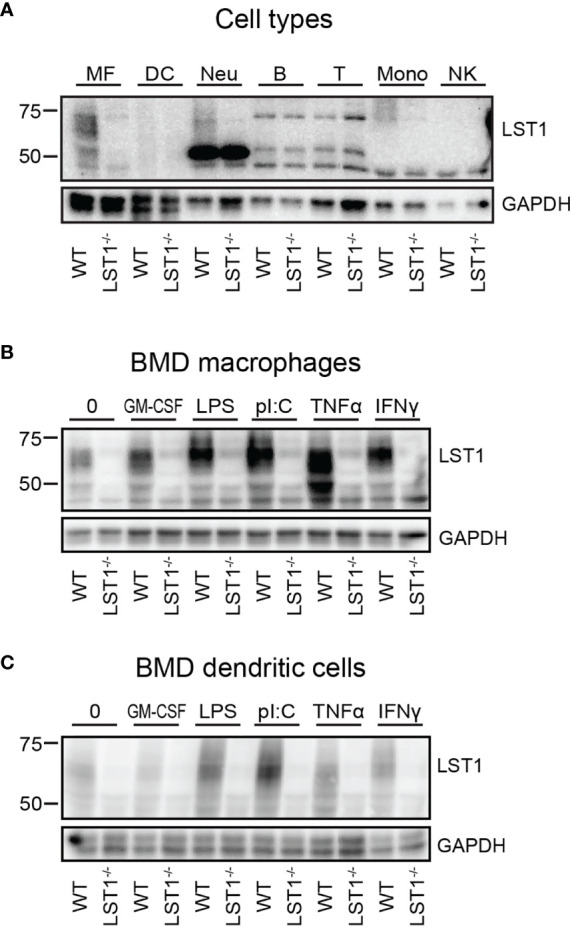
Murine LST1 is expressed in the cells of myeloid lineage and its expression increases after pro-inflammatory stimuli. **(A)** Murine LST1 was detected in total cell lysates by immunoblotting (n=3). MF – bone marrow derived macrophages, DC – bone marrow derived dendritic cells, Neu – Neutrophils, B – B cells, T – T cells, Mono – monocytes, NK – NK cells. Its molecular weight varies between 42 to 71 kDa, likely due to the glycosylation. **(B, C)** Expression of LST1 in bone marrow derived macrophages **(B)** and dendritic cells **(C)** after treatment with various pro-inflammatory stimuli (n=3, see *Materials and Methods* for concentrations).

LST1 is a small transmembrane adaptor protein, which makes homo-dimers *via* cysteine bridges (1). Our previously generated monoclonal antibody LST1/02 to human LST1 (1) did not recognize murine protein. For detection of murine LST1, we have generated a novel monoclonal antibody LST1/06, against recombinant intracellular segment of murine LST1 and verified its specificity (**Figure 1A**). It preferentially binds LST1 under non-reducing conditions, i.e. in its dimeric form, which can be detected on Western blot as a broad double-band of ca 27-37 and 42-71 kDa, depending on the cell type (**Figures 1A, B**, **2**). Lower than expected electrophoretic mobility and presence of N-glycosylation motif (NxS) in the short extracellular sequence suggested that murine LST1 could be glycosylated. Indeed, after addition of glycosylation inhibitor tunicamycin to the BMDM, its apparent molecular mass decreased to ca 29-48 kDa (**Figure 1B**).

### LST1 Protein Levels Are Increased by Pro-Inflammatory Stimuli

The LST1 protein expression analysis using LST1/06 antibody, revealed that LST1 expression in cells that were not activated by proinflammatory stimuli is relatively low. As a result, multiple background bands cross-reacting with LST1/06 antibody, including a band of a similar molecular weight as LST1, became apparent during prolonged Western blot exposures. However, the use of the *Lst1^-/-^* cells as controls allowed us to distinguish the specific signal. Under these conditions, LST1 could be detected in the cells of myeloid lineage, including macrophages (MF), neutrophils (Neu), monocytes (Mon), but not in lymphoid lineage cells (T, B cells and NK cells) (**Figure 2A**). Since it has been observed previously that LST1 expression is elevated by inflammatory stimuli in human cell lines and in inflamed colon tissue (6), we decided to test if LST1 expression is upregulated under inflammatory conditions in leukocytes of myeloid lineage. Indeed, we observed elevated expression of LST1 *in vitro* in murine BMDM and BMDC after overnight (16 hours) stimulation with IFNγ, LPS, TNFα and PolyI:C (**Figures 2B, C**).

### LST1 Deficiency Results in Sex-Dependent Alterations of Trabecular Bone Structure in Mice

**Figure 3:**
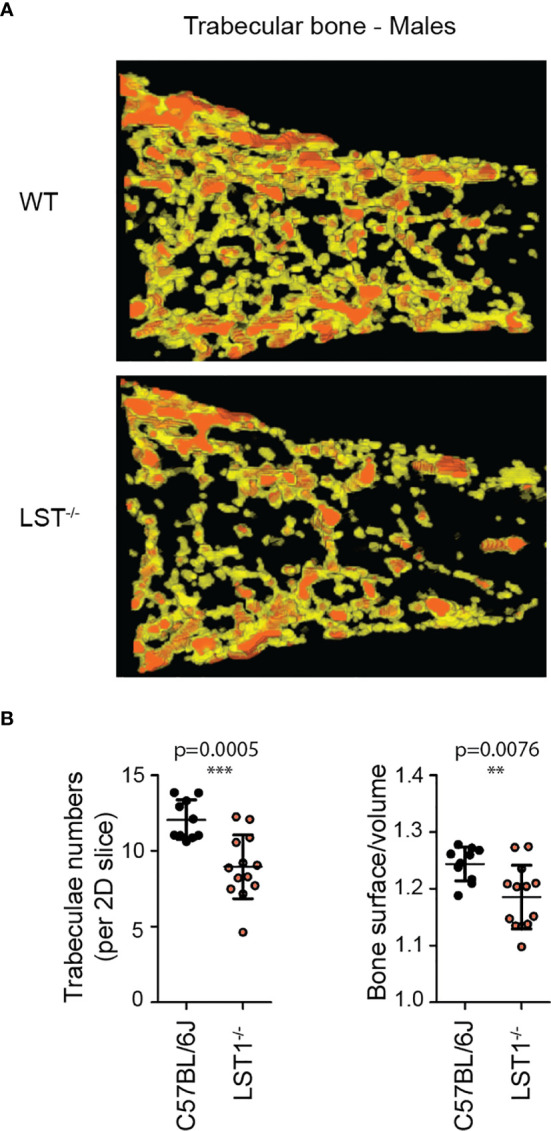
Altered trabecular bone structure in *Lst1^-/-^* mice. **(A)** Representative picture of trabecular bone µCT scan of male bones with diminished numbers of trabeculae in *Lst1^-/-^* animals **(B)** Quantification of trabeculae numbers in 2D plane and ratio between trabecular bone surface and bone volume of WT (n=10) and *Lst1^-/-^* (n=13) mice. Lowered ratio is an evidence of reduced trabeculae segmentation. Dots in scatter plots represent biological replicates (hind limbs, i.e. two dots per mouse). Statistics evaluation was done by Student´s t-test (two-tailed, unpaired) and two-sided Grubb´s test. **p ≤ 0.01, ***p ≤ 0.005.

*Lst1^-/-^* mice were born at standard Mendelian ratios without any manifestation of disease during their aging. The phenotypical analysis of this mouse strain presented on the website of International mouse phenotyping consortium^2^ (17), showed slight increase in the bone mineral density in *Lst1^-/-^* animals, suggesting potential effect on the function of osteoclasts or overall bone homeostasis, which are known to be affected by immune system activity. Though the finding was not considered significant, low p-value associated with the data prompted us to re-evaluate these results. Our analysis by microcomputerized tomography (µCT) did not confirm any differences in the bone mineral density (not shown). On the other hand, it revealed reduced numbers of trabeculae in the trabecular bone tissue of *Lst1^-/-^* mice (**Figures 3A, B**). The trabeculae were also less segmented as demonstrated by the reduced ratio of trabecular bone surface and bone volume (**Figure 3B**). Interestingly, these differences were restricted only to male animals (**Figures 3A,B**; **Supplementary Figure S1**).

### LST1 Deficient Mice Show Alterations in Leukocyte Subset Composition

**Figure 4:**
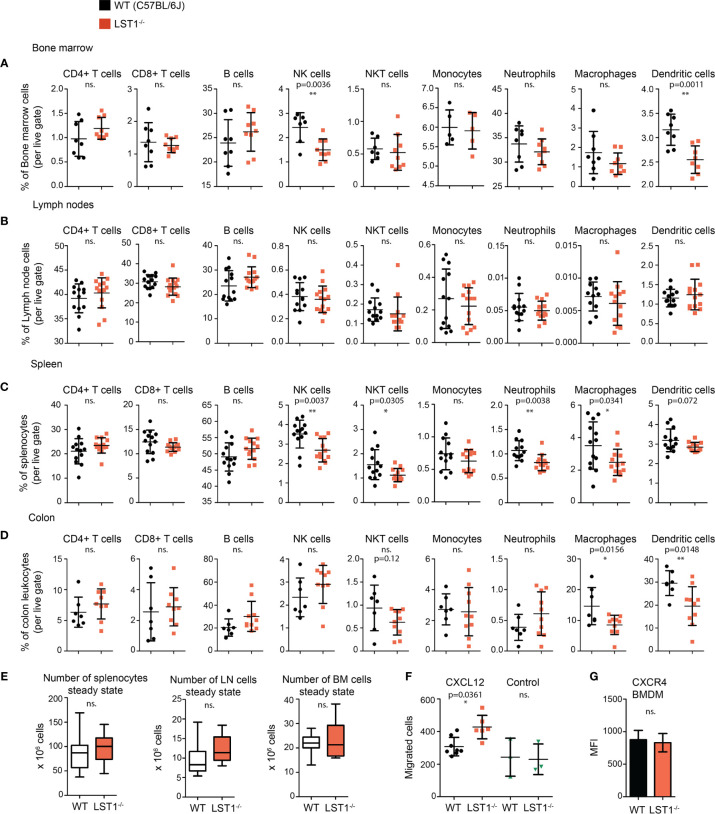
Alterations of leukocyte subset percentages in *Lst1^-/-^* mice. **(A–D)** Steady state percentages of main leukocyte subsets (n=5-15) in the bone marrow **(A)**, lymph nodes **(B)**, spleen **(C)**, and colon **(D)**. Dots in scatter plots represent biological replicates. **(E)** Absolute numbers of cells in steady state spleen, bone marrow and lymph nodes (n=14-25). **(F)** Number of bone marrow derived macrophages (BMDM) that after 2 hours migrated towards CXCL12 (100 ng/ml) (n=6-8). Control samples were without CXCL12 chemo-attractant (n=3). **(G)** Quantification of CXCR4 expression on BMDM surface (n=6). Dots in scatter plots represent biological replicates. Statistics evaluation was done by Student´s t-test (two-tailed, unpaired) and two-sided Grubb´s test. *p ≤ 0.05, **p ≤ 0.01, ns, not significant.

Analysis of steady state lymphocyte populations in *Lst1^-/-^* mice revealed that while B cell and T cell subsets are without change and their percentages are comparable to the wild type mice (WT), other populations show reduced numbers. These include mainly bone marrow and splenic NK cells and splenic NKT cells (**Figures 4A-C**). Among the splenocytes of myeloid lineage, reduced percentages were observed for neutrophils, F4/80^+^ macrophages and to some extent also dendritic cells (**Figure 4C**). Reduced percentages were also observed for dendritic cells in the bone marrow (**Figure 4A**). Importantly, we also observed decrease in macrophage and dendritic cell percentages in the colon (**Figure 4D**). Total cell numbers were not significantly changed between the WT and *Lst1^-/-^* animals in any of the organs analyzed (**Figure 4E**). Reduced percentages of multiple leukocyte subsets could result from defect in cellular migration. To test the migratory capacity of LST1-deficient cells we analyzed chemokine-mediated migration of *Lst1^-/-^* bone marrow-derived macrophages in a transwell assay. Surprisingly, *Lst1^-/-^* cells migrated more efficiently towards CXCL12, ligand of chemokine receptor CXCR4, than WT cells (**Figure 4F**). At the same time CXCR4 expression was not affected by LST1 deficiency (**Figure 4G**). This finding suggests that LST1 negatively regulates CXCR4-mediated migration. It also shows that the migratory capacity of *Lst1^-/-^* cells is not compromised and that there is no general defect in cellular migration that could be responsible for reduced percentages of leukocyte subsets described above.

### Normal Response of *Lst1^-/-^* Mice to LPS Challenge

**Figure 5:**
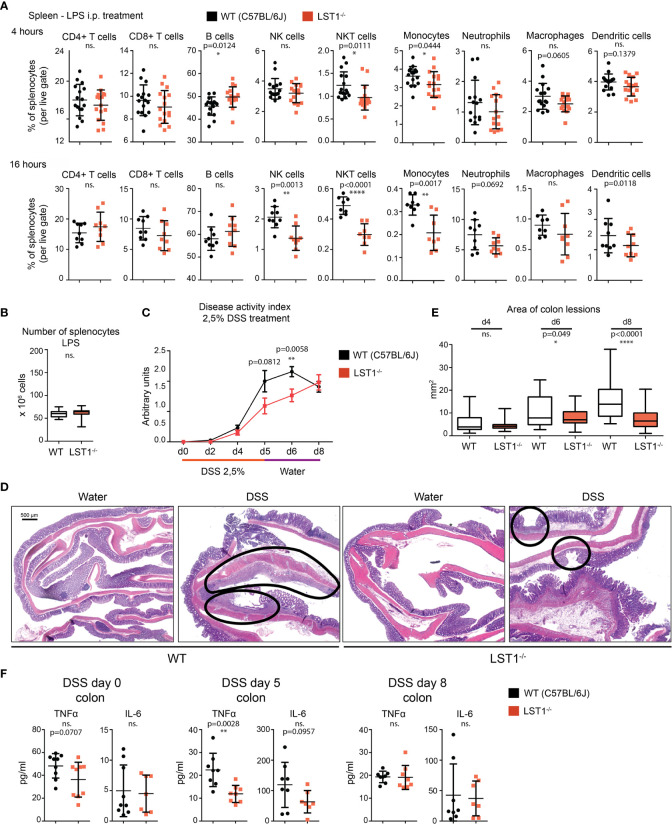
Inflammatory response in *Lst1^-/-^* mice. **(A)** Percentages of major splenocytes subsets 4 and 16 hours after IP injection of LPS (n=8-9). Dots in scatter plots represent biological replicates. **(B)** Spleen cellularity 16 hours after LPS IP injection. (n=10) **(C)** DSS-induced colitis disease activity index (n=6-19 per time point) determined as described in Materials and Methods (higher numbers correspond to more severe symptoms) **(D)** Representative picture of colon sections stained with hematoxylin and eosin from control and DSS challenged WT and *Lst1^-/-^* mice. Lesions are highlighted with black line (100x magnification) **(E)** Quantitative evaluation of the size of colonic lesions induced by DSS treatment during multiple experiments (n=3-5 animals [26-56 lesions] per time point). **(F)** Concentrations of TNFα and IL-6 in colon homogenates from DSS-treated mice. Statistics evaluation was done by Student´s t-test (two-tailed, unpaired) and two-sided Grubb´s test. **p ≤ 0.01, ****p ≤ 0.0001, ns, not significant.

Data showing increase in LST1 expression level after exposure to pro-inflammatory stimuli indicated that LST1 could be involved in the control of inflammation. In addition, we expected that the differences between WT and *Lst1^-/-^* mice will be more apparent under the conditions where LST1 expression is high in WT animals. Therefore, we decided to induce systemic inflammation in WT and *Lst1^-/-^* mice by intraperitoneal injection of LPS. In the spleens of both strains, this resulted in increased percentages of B cells and neutrophils and decreased percentages of other cell populations. When comparing WT and *Lst1^-/-^* mice, majority of the differences and trends observed in the steady state remained preserved or even enhanced. These included mainly reduced percentages of NK and NKT cells, monocytes, dendritic cells and similar (but outside the borderline of statistical significance) reduction in neutrophils (**Figure 5A**). Total numbers of splenocytes remained comparable (**Figure 5B**). In the bone marrow and blood, no additional alterations in *Lst1^-/-^* mice were observed (not shown). TNFα and IL-6 levels in the blood at 4 hours after LPS challenge were also not affected by LST1 deficiency (**Supplementary Figure S2A**). In BMDCs LST1 deficiency did not result in any alteration in LPS-induced cytokine mRNA expression, including IL-1β, IL-6, IL-12, TNFα, IL-15, IL-18, IL-10, and TGFβ (**Supplementary Figure S2B**). Similar results were also obtained in a more limited analysis of BMDM (not shown). In line with these observations, LPS-induced NF-κB activation pathway in BMDM was not affected by LST1 deficiency (**Supplementary Figure S2C**). Moreover, expression of LST1 binding partners SHP1 and SHP2 also was not influenced by LST1 deficiency in LPS-treated BMDM (**Supplementary Figure S2D**) Thus, we concluded that LPS challenge did not reveal any new major differences between leukocyte subset composition and behavior in WT and *Lst1^-/-^* mice.

### *Lst1^-/-^* Mice Show Increased Resistance to Acute Colitis Induced by DSS

Previously published article (6) showed elevated expression of LST1 in inflamed colon tissue from a patient with inflammatory bowel disease (IBD). This disease is characterized by an exaggerated immune response to commensal microbiota and breach of intestinal barrier. However, its etiology is still not completely understood. DSS-induced acute colitis is a widely used mouse model of IBD, where myeloid cells play a major role (18, 19). Due to LST1 expression in the target tissues and our data showing effects of LST1 deficiency on several myeloid populations, we decided to induce DSS colitis in *Lst1^-/-^* male mice to test the effects of LST1 deficiency on the severity of this disease. We used only males for this experiment, since they are more sensitive to DSS treatment (20). This way we reduced variability and a number of animals needed for this experiment and increased probability of detecting differences between WT and *Lst1^-/-^* mice. DSS at concentration 2.5% dissolved in drinking water was administered to the mice for 6 days. Then, DSS solution was changed for plain water for the remaining two days of the experiment (day 7 and 8). Mice were monitored for *Lst1* mRNA expression and for number and area of inflammatory lesions, length of the colon and overall disease activity defined by Disease Activity Index – DAI (an integrated value encompassing weight loss, rectal bleeding and stool consistency). *Lst1* mRNA expression in the colon tissue did not significantly change during the course of the experiment and it was absent from isolated colonic epithelial cells. (**Supplementary Figure S3A**). Disease activity was similar in both WT and *Lst1^-/-^* mice until the day 4 of DSS treatment. However, from the sixth day on, the colitis DAI of *Lst1^-/-^* mice appeared significantly lower than that of WT animals (**Figure 5C**). *Lst1^-/-^* mice displayed less severe rectal bleeding, better stool consistency, and milder colon shortening than WT animals (**Supplementary Figure S3B**). Moreover, areas of damaged colonic epithelium were significantly smaller at days 6 and 8 in the *Lst1^-/-^* mice (**Figures 5D, E**). Expression of pro-inflammatory cytokines detected in colonic lysates was also reduced at day 5 of the experiment (**Figure 5F**). Surprisingly, these differences in colon inflammation did not have an impact on body weight loss, which was similar for both genotypes (**Supplementary Figure S3B**). After changing DSS solution for plain water, WT mice began to recover more rapidly and the DAI reached the same values for both strains at the end of the experiment (**Figure 5C**). The differences in cytokine expression were also no longer detectable at day 8 (**Figure 5F**). On the other hand, colonic lesions in *Lst1^-/-^* animals were still significantly smaller at that time point (**Figure 5E**). Surprisingly, flow cytometry analysis of leukocyte subsets in the colon did not show any striking differences between WT and *Lst1^-/-^* mice. DSS treatment resulted in multiple changes in myeloid immune cells percentages (**Figure 4D**, **Supplementary Figure S3C**). However, they were similar in both genotypes. In the lymphoid compartment, we observed slightly reduced *Lst1^-/-^* T cell percentages on day 5 which then recovered, and increased *Lst1^-/-^* CD4 T cell percentages could be observed on days 6 and 8 (**Supplementary Figure S3C**). Together, these data suggest that LST1 is not just an expression marker in colon samples of IBD patients but likely also an immune system regulator with moderate effects on leukocyte functions, homeostasis and development of colitis.

## Discussion

During the past two decades multiple observations have been made of elevated LST1 mRNA and protein levels under inflammatory conditions and during disease, including IBD, rheumatoid arthritis, and influenza (2, 6–8, 14, 21). Location of LST1 gene in MHCIII locus also suggests function in the regulation of immune response or inflammation. In spite of this, its role in these processes or in leukocyte homeostasis remained largely unknown. In this article, we provide data on LST1 protein expression in mice and show alterations in leukocyte subset composition caused by LST1 deficiency at steady state and under inflammatory conditions. In addition we bring evidence of LST1 effects on the severity of experimentally induced acute colitis.

Expression of LST1 protein has previously been analyzed in human cells and tissues with conflicting results. Monoclonal antibody LST1/02 recognizing human but not murine LST1, which was developed in our laboratory, revealed expression pattern largely restricted to the leukocytes of myeloid linage (1), while antibody developed by Schiller et al. showed broad expression pattern including expression outside hematopoietic system (3, 6). Here we describe another monoclonal antibody LST1/06, which recognizes murine LST1. It was generated separately from LST1/02 after immunization with recombinant protein comprising the entire cytoplasmic part of the murine LST1 and its specificity was validated on HEK293 transfectants expressing the murine LST1 (data not shown) and on LST1-deficient mice. The intensity of signals we obtain with LST1/06 antibody on Western blots is generally lower than the intensities we typically observe when using LST1/02 antibody on human samples. However, the reactivities of both antibodies are very similar, showing expression pattern restricted to myeloid cells and an absence in lymphocytes [**Figure 2A** and (1)]. Publically available data on human LST1 mRNA expression from Human Protein Atlas (22) and on mouse LST1 mRNA from ImmGen consortium (23) both support conclusions reached with our antibodies showing myeloid cell-restricted expression pattern, though low level of expression in other tissues cannot be completely excluded. Consistently with already published data (2, 6), pro-inflammatory stimuli enhanced expression of LST1 in myeloid cells.

LST1 deficiency in the mouse influences leukocyte homeostasis. We observed decreased percentages of myeloid cells in *Lst1^-/-^* mice. Significant reduction or at least a similar tendency were almost universally observed for majority of analyzed myeloid cell subsets in the bone marrow, spleen and colon, though the changes were usually relatively modest (**Figures 4A–D**). Some of the possible explanations of this phenotype include differences in chemokine receptor expression and differences in migration ability. We have analyzed expression of chemokine receptors CCR5, CCR6 and CXCR4 on leukocyte subsets in the spleen, bone marrow and colon at steady state and after DSS treatment, but we did not observe any significant differences between WT and *Lst1^-/-^* animals that could explain alterations in frequencies of these subsets (not shown). Intriguingly, *Lst1^-/-^* BMDM showed enhanced migration towards CXCL12. While this observation is interesting, it does not show any attenuation of migratory capacity and, as a result, cannot explain the reduced percentages of macrophages and other cell types in the lymphoid organs analyzed in this study. Reduced percentages were frequently observed also for LST1 non-expressing NK and NKT cells (**Figures 4A, C**). Since NK and NKT cells do not express LST1 the effect on these cells must be indirect, possibly mediated by myeloid cell-derived cytokines or other factors. We have tested mRNA expression of several cytokines that could affect NK cell numbers, including IL-15, IL-18, and IL-12 in *Lst1^-/-^* BMDCs and BMDMs. However, their levels were not altered in these cells (**Supplementary Figure S2B** and not shown). It is also possible that other cytokines were involved or that the NK cell supporting cytokine production *in vivo* was attenuated due to the diminished numbers of myeloid cells producing these cytokines rather than due to the reduction in cytokine expression levels.

Immune system of the gut and gut microbiota are known to have effects on bone homeostasis (24, 25). Germ-free mice were shown to have increased trabecular bone volume while gut dysbiosis after antibiotic treatment results in reduction in trabecular bone volume (26, 27). One could speculate that effects of LST1 on the activity of the immune system of the gut are linked to the alterations in trabecular bone structure observed in the present study. The experiments with the mouse model of DSS induced colitis suggested altered response to intestinal microbiota in *Lst1^-/-^* mice. On the other hand, we have not detected any changes in pro-inflammatory cytokine production in the steady state gut in *Lst1^-/-^* mice that could be a sign of altered activity of the gut immune system. In addition, during DSS-induced colitis, LST1 deficiency had mitigating effects on gut inflammation further arguing against the possibility that the lack of LST1 in the gut promoted inflammation-driven changes in trabecular bone structure. Chronic inflammation in other parts of the body could potentially also affect various parameters of the bone. However, so far we have not found any evidence of chronic inflammation in *Lst1^-/-^* mice. Finally, it is also possible that LST1 deficiency in the osteoclasts leads to alterations in their function and defects in trabecular bone formation. Currently available data do not provide sufficient support for any of these explanations and further research is needed to clarify this issue.

A number of polymorphisms have been detected in human *LST1* gene (5). Some of these polymorphisms, thought to affect mainly *LST1* gene expression and splicing, are associated with inflammatory conditions such as psoriasis, nephritis in systemic lupus erythromatosus and rheumatoid arthritis, or graft versus host disease severity (5). Moreover, expression of LST1 is increased in rheumatoid arthritis patients (7) and in colon samples from patients with inflammatory bowel disease (IBD) (6). These connections to IBD and various other inflammatory conditions, prompted us to test the effects of LST1 deficiency on inflammatory response and, in particular, on DSS-induced colitis in mice. DSS-induced collitis is one of the most widely used models of human IBD (28). Its overall symptoms and course are similar to this disease (19). DSS ingested by experimental animals with drinking water is cleaved into smaller fragments, penetrates colon barrier and dissolves the mucus layer. This leads to infiltration, colonization and damaging of colon epithelium by bacteria and viruses. Colonization of colon epithelia by bacteria is followed by infiltration of myeloid immune cells (neutrophils, dendritic cells, monocytes), causing acute colon inflammation and its symptoms (blood in the stool, colon shortage, diarrhea). Lymphoid cells (T, B, NK cells) and their interplay with myeloid immune cells also showed important impact on the severity of acute colitis (29–35).

Strikingly, *Lst1^-/-^* mice showed milder and delayed course of the disease. Disease Activity Index was significantly lower on the 6^th^ day of DSS treatment. These results were also confirmed by histology, which showed less severe destruction of colon epithelium (**Figures 5D, E**). In the final part of the experiment, after changing DSS solution for plain water on day 6, DAI of WT animals improved while in the *Lst1^-/-^* mice DAI kept rising even after DSS solution was changed for plain water. On day 8, DAI reached the same severity as already improving WT mice. Data from mouse and patient samples show that resident and newly infiltrating myeloid immune cells are responsible for the first wave of immune response during DSS induced colitis (36). Reduced myeloid cell percentages in *Lst1^-/-^* colon and lymphatic tissue might explain the slower kinetics of disease development. On the other hand, LST1 may also be contributing to the resolution of inflammation. This could explain why the DAI in *Lst1^-/-^* mice was still raising after day 6, when the WT mice were already in the process of healing. However, there are currently no data addressing the role of LST1 in the resolution of inflammation. Additional possibilities include direct effects of LST1 expressed by epithelial cells on their barrier function or compensatory changes in SHP1 and SHP2 expression in myeloid cells. However, we could not detect any *Lst1* mRNA in colonic epithelial cells nor any compensatory changes in SHP1/2 expression in LPS-treated BMDM. On the other hand, we observed significant reduction in TNFα expression in the gut on day 5 of DSS colitis and a similar trend for IL-6. TNFα is known to be involved in the pathogenesis of IBD and can be upstream of IL-6 expression (37). It is produced not only by myeloid cells, but also by Th1 cells. Myeloid cell numbers in the gut and their ability to produce TNFα *in vitro* were not affected by LST1 deficiency. On the other hand, overall T cell numbers were reduced in the gut on day 5 of DSS colitis. Since T cells do not express LST1, their reduced numbers could be the result of altered chemokine expression by myeloid cells or altered kinetics of dendritic cell-mediated antigen presentation, T cell activation or differentiation in *Lst1^-/-^* mice. Any of these changes could lead to transient reduction in Th1 cell numbers in the gut and reduced TNFα production. However, these hypotheses have to be further elucidated in future studies. Molecules and molecular mechanisms affecting the course and outcome of DSS-induced collitis often play a role in human IBD (19). Our results suggest that defects in LST1 expression and LST1 polymorphisms should be considered in future studies of this and related diseases in humans.

## Data Availability Statement

The raw data supporting the conclusions of this article will be made available by the authors, without undue reservation.

## Ethics Statement

The animal study was reviewed and approved by Animal Care and Use Committee of the Institute of Molecular Genetics.

## Author Contributions

MF and JT with contribution from NP, JK, JB and TS performed majority of experiments and data analysis. JT, JB, KV and MF performed analysis of mouse colitis model. NP carried out analysis of LST1 and SHP1/2 expression analysis and ELISA assays. MF, JK, TS and NP performed flow cytometry analysis of healthy mice. FS and JPr carried out μCT analysis. JPo and PA generated LST1/06 hybridoma and corresponding monoclonal antibody. TB, JPr and RS contributed to experimental design and data analysis. BM assisted with *Lst1^-/-^* mice rederivation and preliminary phenotype analysis. TB, BM and RS secured funding and supervised the study. TB and MF wrote the paper. All authors contributed to the article and approved the submitted version.

## Funding

This study was supported by Czech Science Foundation (GACR, project P302-12-G101) and by institutional funding from Institute of Molecular Genetics, CAS (RVO68378050). The results were obtained using the research infrastructure of the Czech Center for Phenogenomics supported by the projects of the Ministry of Education, Youth and Sports (MEYS) LM2018126 and OP RDI CZ.1.05/2.1.00/19.0395, and CZ.1.05/1.1.00/02.0109 provided by the MEYS and ERDF, and OP RDE CZ.02.1.01/0.0/0.0/16_013/0001789 by MEYS and ESIF. Centre d’Immunophénomique is supported by the Investissement d’Avenir program PHENOMIN (French National Infrastructure for mouse Phenogenomics; ANR-10-INBS-07).

## Conflict of Interest

The authors declare that the research was conducted in the absence of any commercial or financial relationships that could be construed as a potential conflict of interest.

## Footnotes

1
https://www.mousephenotype.org/impress/ProcedureInfo?action=list&procID=1225&pipeID=7

2
https://www.mousephenotype.org/data/genes/MGI:1096324
