# Supplementary material for: Regulation of Inflammatory Response by Transmembrane Adaptor Protein LST1
Source: Front Immunol. 2021 Apr 27;12:618332. doi: 10.3389/fimmu.2021.618332 (PMC8111073; doi:10.3389/fimmu.2021.618332)
Supplement: Supplementary file 1 [file DataSheet_1.pdf]

Supplementary Material

Supplementary Figures

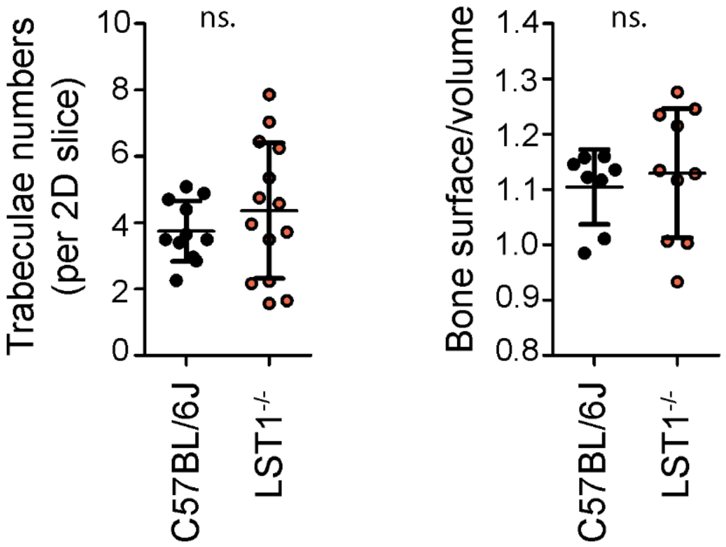

Supplementary figure S1. Trabecular bone structure in *Lst1*$^{-/-}$ female mice. Quantification of trabeculae numbers in 2D plane and ratio between trabecular bone surface and bone volume did not show any differences between female WT and *Lst1*$^{-/-}$ (n=8-14) mice. Statistics evaluation was performed by Student´s t-test (two-tailed, unpaired) and two-sided Grubb´s test.

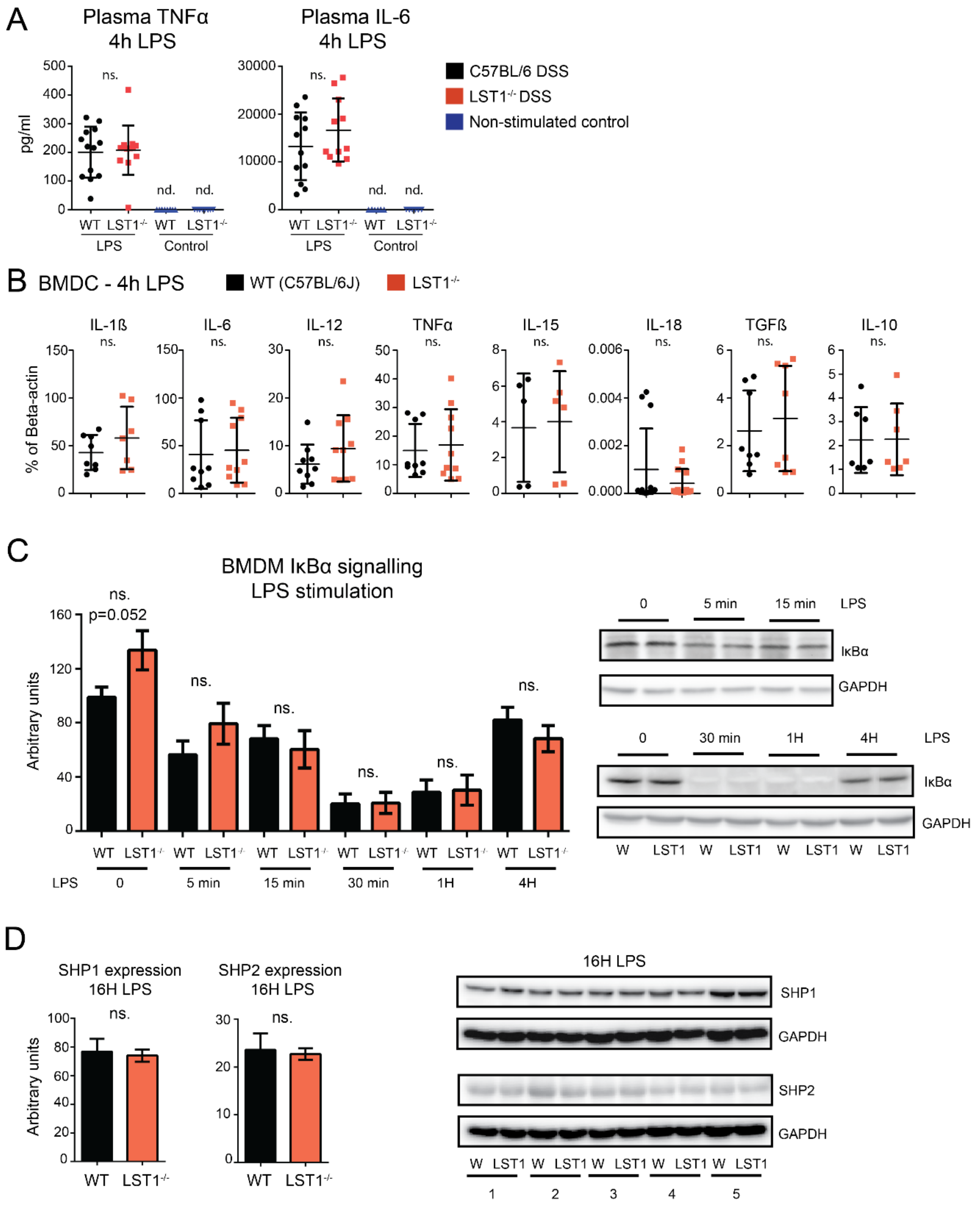

Supplementary figure S2. Inflammatory response in LST1$^{-/-}$ mice. (**A**) TNFα and IL-6 levels in plasma measured by ELISA 4 hours after intraperitoneal LPS injection (n=11-13) (**B**) Cytokine mRNA expression measured by quantitative real-time PCR in BMDC after 4 hours of stimulation with LPS (100 ng/ml), expression was normalized to β-actin. (n=5-13) (**C**) IκBα degradation in BMDM stimulated with LPS (100 ng/ml). Quantification and representative immunoblot (n=4-9) (**D**) Expression of SHP1 and SHP2 phosphatases in BMDM after 16 hour incubation with LPS (100 ng/ml) (n=5). Statistics evaluation was done by Student´s t-test (two-tailed, unpaired), One-way ANOVA with Tukey post test and two-sided Grubb´s test.

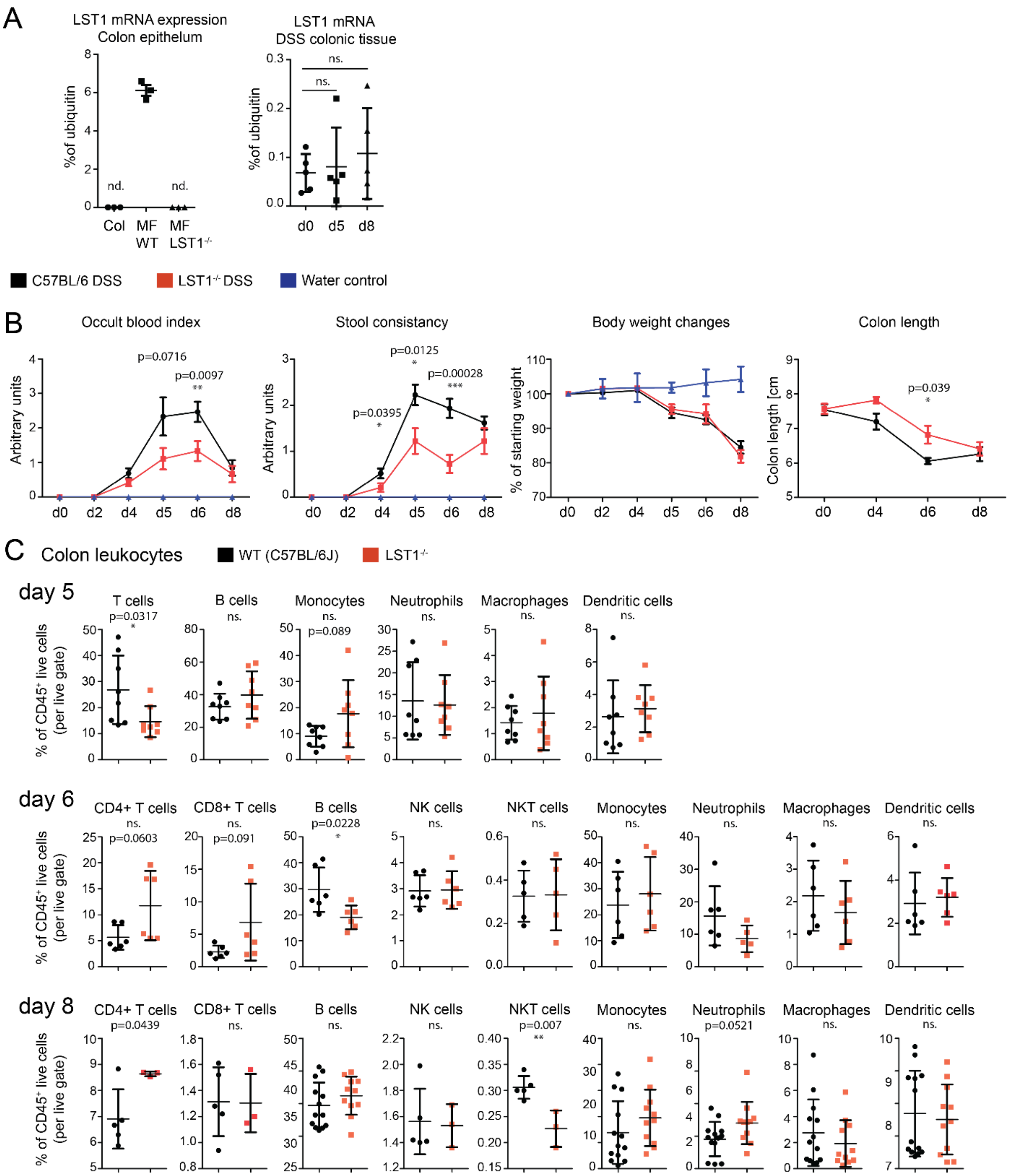

Supplementary figure S3. DSS-induced colitis in LST1$^{-/-}$ mice. (**A**) Expression of LST1 mRNA in colon epithelium cells compared to the WT macrophages and LST1$^{-/-}$ macrophages (n=2, technical triplicates from representative experiment are shown), and of LST1 mRNA in colonic tissue from DSS-treated mice (n=5), expression was normalized to ubiquitin. (**B**) Parameters used for calculation of disease activity index in Figure 5C and changes in colon length, an additional parameter characterizing the disease severity (n=5-20). (**C**) Leukocyte subsets present in the colon at day 5, 6 and day 8 after the initiation of DSS treatment (n=3-13). Statistics evaluation was done by Student´s t-test (two-tailed, unpaired), One-way ANOVA with Tukey post test and two-sided Grubb´s test.

Supplementary tables

Supplementary Table S1. List of flow cytometry antibodies

Marker	Fluorophore – Company - Clone	Fluorophore – Company - Clone
**CD8a**	PE-CF594 – BD Biosciences – 53-6.7	FITC – ExBio – 53-6.7
**NK1.1**	APC – BD Biosciences – PK136	PB – Biolegend – PK136
**Ly6G**	BV421 – BD Biosciences – 1A8	e450 – Biolegend – 1A8
	BV510 – Biolegend – 1A8	
**CD19**	BV510 – BD Biosciences – 1D3	APC – Biolegend – 605
**Ly6C**	FITC – BD Biosciences – AL-21	PE-Cy7 – Biolegend – HK1.4
**CD11b**	PerCP-Cy5.5 – Biolegend – M1/70	PE – Biolegend – M1/70
**F4/80**	PE-Dazzle594 – Biolegend – BM8	A488 – Biolegend – BM8
**CD11c**	PE-Cy7 – BD Biosciences – HL3	APC – Biolegend – N418
**Fc block**	2.4g2 supernatant (produced in lab.)	2G4 – BD Biosciences
**CD45**	A700 – Biolegend – 30-F11	APC – Biolegend – 104
**CD44**	PE – Biolegend – IM7	
**CD49**	BV510 – BD Biosciences – R1-2	
**CD5**	BV421– BD Biosciences – 53-7.3	
**CD4**	FITC – BD Biosciences – RM4-5	
**Klrg1**	PerCP-Cy5.5 – Biolegend – 2F1/KLRG1	
**γδTCR**	BV605 – BD Biosciences – GL3	
**GITR**	BV711 – BD Biosciences – DTA-1	
**CD25**	PE-CY7 – BD Biosciences – PC61	
**CD62L**	APC-CY7– BD Biosciences – MEL14	
**CD21/CD35**	PE – BD Biosciences – 7G6	
**Bst2**	BV605 – Biolegend – 927	
**CXCR4**	APC – Biolegend – L276F12	
**CCR5**	PE – Biolegend – HM-CCR5	
**CCR6**	BV785 – Biolegend – 29-2L17	
**CCR7**	APC-eFluor 780 – eBioscience – 4B12	
**sCD23**	BV711 – BD Biosciences – B3B4	
**MHCII**	APC-Cy7 – Biolegend – M5/114.15.2	
**EpCAM**	APC – Biolegend - G8.8	
**Ghost Dye™**	UV450 – Tonbo biosciences –13-0868	

Supplementary Table 2. List of qRT-PCR primers

	Forward	Reverse
**IL-1β**	TGT-AAT-GAA-AGA-CGG-CAC-ACC	TCT-TCT-TTG-GGT-ATT-GCT-TGG
**IL-6**	TGA-TGG-ATG-CTA-CCA-AAC-TGG	TTC-ATG-TAC-TCC-AGG-TAG-CTA-TGG
**IL-10**	CAG-AGC-CAC-ATG-CTC-CTA-GA	TGT-CCA-GCT-GGT-CCT-TTG-TT
**IL-12**	CCA-GGT-GTC-TTA-GCC-AGT-CC	GCA-GTG-CAG-GAA-TAA-TGT-TTC-A
**IL-15**	AAC-AGC-TCA-GAG-AGG-TCA-GGA	CCA-TGA-AGA-GGC-AGT-GCT-TT
**IL-18**	GAC-AAC-ACG-CTT-TAC-TTT-ATA-CGG	CAG-TGA-AGT-CGG-CCA-AAG-TT
**TNFα**	TCT-TCT-CAT-TCC-TGC-TTG-TGG	GGT-CTG-GGC-CAT-AGA-ACT-GA
**TGFβ**	TGG-AGC-AAC-ATG-TGG-AAC-TC	CAG-CAG-CCG-GTT-ACC-AAG
**Beta-actin**	GAT-CTG-GCA-CCA-CAC-CTT-CT	GGG-GTG-TTG-AAG-GTC-TCA-AA
**Ubiquitin**	ATG-TGA-AGG-CCA-AGA-TCC-AG	TAA-TAG-CCA-CCC-CTC-AGA-CG
**LST1**	CTG-ATG-ACA-ATG-GGA-TCT-GGT	CAG-GAT-GAT-GAC-AAG-CAG-GA
